# Controlled impact evaluation of a birth registration intervention, Burkina Faso

**DOI:** 10.2471/BLT.18.221705

**Published:** 2019-02-25

**Authors:** Evelina Martelli, Maria Castiglioni, Gianpiero Dalla-Zuanna, Leonardo Emberti Gialloreti, Colette Guiebre, Honorine Medah Dabiret, Adriana Gulotta, Angela Silvestrini, Francesco Di Domenicantonio, Palmira Gianturco, Maria Cristina Marazzi

**Affiliations:** a Birth Registration for All Versus Oblivion (BRAVO), Community of Sant’Egidio, Piazza di S. Egidio 3/a, 00153 Rome, Italy.; b Department of Statistical Sciences, Padua University, Padua, Italy.; c Department of Biomedicine and Prevention, Tor Vergata University of Rome, Rome, Italy.; d Birth Registration for All Versus Oblivion (BRAVO), Community of Sant’Egidio, Ouagadougou, Burkina Faso.; e Ministry of Justice, Human Rights and Civic Promotion, Ouagadougou, Burkina Faso.; f Office for Demographic Structure and Dynamics, Italian National Institute of Statistics, Rome, Italy.; g Department of Social Policies, Municipality of Rome, Rome, Italy.; h Department of Human Sciences, Libera Università Maria SS. Assunta University, Rome, Italy.

## Abstract

**Objective:**

To evaluate the impact of the introduction of secondary civil registration centres on birth registrations within 60 days of birth, in Burkina Faso.

**Methods:**

The faith-based organization Sant’Egidio supported the inauguration of secondary birth registration centres within seven health centres in Réo from July 2015 and four health centres in Godyr from February 2015, at which delivery and vaccination services were available. We calculated the number of timely registrations per 1000 population before and after the launch of the intervention in both the intervention and control municipalities. We used a logistic regression model to evaluate the probability of non-registration as a function of the health centre services used and various demographic and health characteristics, obtained through health registers data and interviews.

**Findings:**

Compared with the previous 12 months, the number of timely birth registrations in Réo and Godyr rose from 502 to 2094 (317.1%) and from 267 to 793 (197.0%) during the first 12 months of the intervention. In the two control municipalities, the numbers were unchanged. Infants whose mothers attended health centres for delivery, but did not return for vaccinations, had the lowest proportions of birth registration (69.0%; 294/426; in Réo and 70.2%; 40/57 in Godyr). Infants of mothers who were not interviewed were more likely to not having a timely birth registration (in Réo odds ratio, OR: 6.25; 95% confidence interval, CI: 4.10–9.52 and in Godyr OR: 25.64; 95% CI: 4.31–166.67).

**Conclusion:**

Introduction of secondary registration centres within health centres increased timely birth registrations.

## Résumé

**Objectif:**

Évaluer l'incidence de l'introduction de centres secondaires d'enregistrement des déclarations d'état civil sur l'enregistrement des naissances en temps voulu (dans les 60 jours après la naissance) au Burkina Faso.

**Méthodes:**

L'organisation religieuse Sant’Egidio a soutenu l'inauguration de centres secondaires d'enregistrement des naissances au sein de sept centres de santé à Réo en juillet 2015 et au sein de quatre centres de santé à Godyr en février 2015; des centres qui proposaient des services d'accouchement et de vaccination. Nous avons calculé le nombre d'enregistrements réalisés en temps voulu, pour 1 000 habitants, avant et après le lancement de cette initiative, tant dans les municipalités ciblées par l'intervention que dans des municipalités témoins. Nous avons utilisé un modèle de régression logistique pour évaluer la probabilité d'un non-enregistrement de la naissance en fonction des services utilisés dans les centres de santé et de diverses caractéristiques démographiques et de santé, à partir des données obtenues dans les registres médicaux et au cours d'entretiens.

**Résultats:**

Comparativement aux 12 mois antérieurs, à Réo et à Godyr, le nombre d'enregistrements en temps voulu des naissances a augmenté durant les 12 premiers mois de l'intervention, en passant de 502 à 2 094 (317,1%) et de 267 à 793 (197,0%) respectivement. Dans les deux municipalités témoins, les chiffres n'ont pas évolué. Les plus faibles proportions d'enregistrement ont correspondu aux enfants nés de mères s'étant rendues dans les centres de santé pour accoucher, mais sans y revenir pour des services de vaccination (à Réo: 69,0%; 294/426 et à Godyr: 70,2%; 40/57). Les naissances d'enfants nés de mères n'ayant pas été vues en entretien ont été plus susceptibles de ne pas être enregistrées en temps voulu (à Réo: rapport des cotes (RC) : 6,25; intervalle de confiance (IC) de 95%: 4,10–9,52; et à Godyr: RC: 25,64; IC 95%: 4,31-166,67).

**Conclusion:**

L'introduction de centres secondaires d'état civil au sein des centres de santé a permis d'augmenter le nombre d'enregistrements des naissances réalisés en temps voulu.****

## Resumen

**Objetivo:**

Evaluar el impacto de la introducción de centros secundarios de registro civil para la inscripción oportuna (en los 60 días siguientes al nacimiento) de los nacimientos en Burkina Faso.

**Métodos:**

La organización religiosa Sant’Egidio apoyó la inauguración de centros secundarios de registro de nacimientos en siete centros de salud en Réo desde julio de 2015 y cuatro centros de salud en Godyr desde febrero de 2015, en los que se disponían de servicios de parto y vacunación. Se ha calculado el número de registros oportunos por cada 1000 habitantes antes y después del lanzamiento de la intervención tanto en los municipios intervenidos como los de control. Se ha seguido un modelo de regresión logística para evaluar la probabilidad del no registro como una función de los servicios de los centros de salud utilizados y diferentes características demográficas y de salud, obtenidas a través de los registros de datos de salud y entrevistas.

**Resultados:**

Comparados con los 12 meses anteriores, el número de registros de nacimientos oportunos en Réo y Godyr aumentó de 502 a 2094 (317,1 %) y de 267 a 793 (197,0 %) durante los primeros 12 meses de la intervención. Las cifras no cambiaron en los dos municipios de control. Los bebés cuyas madres asistieron a los centros de salud para el parto, pero no regresaron para recibir las vacunas tuvieron las proporciones más bajas de registros de nacimientos (69,0 %; 294/426 en Réo y 70,2 %; 40/57 en Godyr). Los bebés de madres que no fueron entrevistadas tuvieron más probabilidades de no ser inscritos oportunamente en el registro de nacimientos (en la razón de probabilidades de Réo, RP: 6,25; intervalo de confianza del 95 %, IC: 4,10-9,52 y en Godyr, RP: 25,64; IC del 95 %: 4,31-166,67).

**Conclusión:**

La introducción de centros secundarios de registro en los centros de salud aumentó los registros de nacimientos oportunos.

## ملخص

**الغرض:**

تقييم أثر افتتاح مراكز التسجيل المدني الثانوية، على تسجيل المواليد في بوركينا فاصو في الوقت المناسب (في غضون 60 يوماً من الولادة).

**الطريقة:**

دعمت المنظمة الدينية Sant’Egidio افتتاح مراكز تسجيل المواليد الثانوية في سبعة مراكز صحية في "ريو" من تموز/يوليو 2015، وأربعة مراكز صحية في "جودير" ابتداءً من شباط/فبراير 2015، حيث تتوفر خدمات الولادة والتطعيم. لقد قمنا باحتساب عدد التسجيلات في الوقت المناسب لكل 1000 نسمة من السكان قبل وبعد بدء التدخل في كل من بلديات التدخل والتحكم. استخدمنا نموذج التحوّف اللوجيستي لتقييم احتمال عدم التسجيل كوظيفة لخدمات المركز الصحي المستخدمة، ومختلف الخصائص الديموغرافية والصحية، التي تم الحصول عليها من خلال بيانات السجلات الصحية والمقابلات الشخصية.

**النتائج:**

مقارنة مع الأشهر الـ 12 السابقة، ارتفع عدد تسجيلات المواليد في الوقت المناسب في "ريو" و"جودير" من 502 إلى 2094 (317.1٪)، ومن 267 إلى 793 (197.0٪)، خلال الأشهر الـ 12 الأولى من التدخل. في كل من البلديتين اللتين خضعتا للضبط، كانت الأرقام دون تغيير. وكان الرضع الذين حضرت أمهاتهم في مراكز صحية للولادة، ولكن لم يعدن للحصول على التطعيمات، كان للتطعيم لديهم أدنى نسب تسجيل للمواليد (69.0٪؛ و294/426؛ في "ريو" و70.2٪؛ 40/57 في "جودير"). الرضع الذين لم تحضر أمهاتهم مقابلات شخصية، كانوا أكثر عرضة لعدم تسجيل المواليد في الوقت المناسب (نسبة الاحتمالات في "ريو"، بنسبة أرجحية: 6.25؛ فاصل الثقة 95٪: 4.10 إلى 9.52 في "جودير" بنسبة أرجحية: 25.64؛ ‏بنسبة أرجحية 95٪: 4.31 إلى 166.67).

**الاستنتاج:**

أدى افتتاح مراكز التسجيل الثانوية داخل المراكز الصحية إلى زيادة تسجيل المواليد في الوقت المناسب. ****

## 摘要

**目的:**

旨在评估在布基纳法索设立二级民事登记中心对及时（出生后 60 天内）出生登记的影响。

**方法:**

以信仰为基础的非营利组织圣艾智德团体从 2015 年 7 月起在雷奥县的七个健康中心设立二级出生登记中心，以及从 2015 年 2 月起在 Godyr 县的四个健康中心设立二级出生登记中心，以提供分娩和疫苗接种服务。我们计算了干预和控制城市在发起干预前后每 1000 人中进行及时登记的人数。我们使用逻辑回归模型来评估未登记的概率，根据所使用的健康中心服务以及健康登记数据和访谈获得的各种人口统计和健康特征的函数。

**结果:**

与前 12 个月相比，雷奥县和 Godyr 县的及时出生登记人数分别从干预前的 502 人增加到 2094 人 (317.1％) 和从 267 增加到 793 人 (197.0％)。在两个控制城市中，人数没有发生变化。母亲在健康中心进行分娩但未接种疫苗的婴儿出生登记比例最低（雷奥县为 69.0％；294/426；Godyr 县为 70.2%；40/57）。其母亲未接受访谈的婴儿更有可能没有及时进行出生登记（雷奥县的优势率，OR：6.25；95% 置信区间，CI：4.10–9.52 以及 Godyr 县的 OR：25.64，95％ CI：4.31-166.67）。

**结论:**

在健康中心内设立二级登记中心可以增加及时出生登记的人数。****

## Резюме

**Цель:**

Оценка эффекта внедрения вспомогательных центров регистрации актов гражданского состояния на своевременную (до 60 дней с момента рождения) регистрацию новорожденных в Буркина-Фасо.

**Методы:**

Религиозное сообщество «СантЭгидио» (Sant’Egidio) поддержало внедрение вспомогательных центров регистрации рождений в семи центрах здравоохранения в Рео с июля 2015 года и в четырех центрах здравоохранения в Годыре с февраля 2015 года, предоставляющих услуги по родовспоможению и вакцинации. Авторы рассчитали количество своевременных регистраций на 1000 человек населения до и после принятия данных мер в охваченных ими муниципалитетах и в контрольных муниципалитетах. Использовалась модель логистической регрессии для оценки вероятности отсутствия регистрации как функции использованных услуг центров здравоохранения, а также разнообразных демографических показателей и показателей здоровья, полученных через органы здравоохранения и путем опросов.

**Результаты:**

В сравнении с предыдущими 12 месяцами количество своевременно зарегистрированных рождений в Рео и Годыре выросло с 502 до 2094 (317,1%) и с 267 до 793 (197,0%) за первые 12 месяцев осуществления мероприятий. В двух контрольных муниципалитетах цифры остались без изменений. Дети, чьи матери пользовались услугами медицинских центров для родов, но не вернулись для вакцинации, имели самую низкую долю регистрации рождений (69,0% или 294/426 в Рео и 70,2% или 40/57 в Годыре). Дети матерей, не участвовавших в опросе, с большей вероятностью не были зарегистрированы своевременно (отношение шансов для Рео составило 6,25; 95%-й ДИ: 4,10–9,52, а для Годыра 25,64; 95%-й ДИ: 4,31–166,67).

**Вывод:**

Ввод в действие дополнительных центров регистрации в медицинских центрах увеличил процент своевременной регистрации новорожденных.****

## Introduction

Civil registration records the occurrence and characteristics of vital events, providing individuals with legal proof of identity and family relationships, and protecting children from exploitation.^1^^–^^6^ Having a birth registration grants legal protection and fundamental rights, especially for the most vulnerable children, such as those belonging to ethnic minorities or who have been orphaned.^7^ The registration facilitates the production of vital statistics, the measurement of fertility and mortality, the monitoring of population movements,^8^^,^^9^ and the development of data-driven policies and interventions.^5^^,^^6^ Studies have shown that a direct correlation exists between the presence of a well-functioning civil registration and vital statistics system within a country and the health outcomes of its population.^8^^,^^10^^–^^12^ Further, timely registration (defined in Burkina Faso law as within 60 days of birth) is a prerequisite for tracing and recording neonatal and infant deaths.^5^^,^^13^ Civil registration is also more accurate and cost–effective than other statistical sources.^14^^–^^16^ Although analysis based on data from Demographic and Health Surveys or Multiple Indicator Cluster Surveys^11^^,^^17^^–^^19^ and cross-sectional studies can determine the proportion of registered individuals,^20^^–^^24^ such surveys and studies cannot identify interventions to improve this proportion.

In the sub-Saharan country of Burkina Faso, each municipality has a principal civil registration centre and vital events can only be recorded in the municipality in which the event occurred. The mayor is permitted to establish secondary civil registration centres in villages and health centres, but these are rare. Births and deaths can be registered for free within 60 days of their occurrence. Event registration after this period is only possible in principal registration centres after a judicial procedure and payment of a fine of about 4 United States dollars.^25^^,^^26^ Despite these penalties, the registrations of births in Burkina Faso and in most sub-Saharan African countries are predominantly late;^2^^,^^27^ by examining a sample of registers, we identified that about 80% of birth registrations during a 4-month period in 2014 took place more than 60 days after birth (Martelli E et al., Sant’Egidio, unpublished data, 2014).

To promote civil registration, Sant’Egidio, an international faith-based organization, launched the Birth Registration for All Versus Oblivion (BRAVO) programme in 2008. The programme is currently active in Burkina Faso, Malawi and Mozambique.^7^^, ^^28^ In Burkina Faso, Sant’Egidio birth registration programme advised and supported the national registration campaign in 2009. During the campaign, teams of civil servants visited all villages and offered free registrations. In 2010, just before the end of this campaign, 52.3% (7691/14 704) of children aged 0–4 years had a birth certificate.^18^ In 2014, this proportion had increased to 59.9% (7791/13 006).^17^

To further promote the timely civil registration of births, the Sant’Egidio birth registration programme cooperated with the local authorities to set up secondary civil registration centres in health centres at which deliveries and vaccinations take place. Here, we present the results of a 2-year impact evaluation of this intervention, by estimating the number of timely birth registrations and, by considering several possible factors that may facilitate or hamper registrations.

## Methods

### Study setting

The Centre-Ouest region of Burkina Faso was selected for the location of the intervention for its below-average proportion of registered births. In 2010 the proportion of children younger than 5 years whose birth had been registered was 62.4% (725/1162; national: 76.9%; 11 307/14 704).^18^ The average number of children per woman in this region was 6.6 (national: 6.0) and infant mortality rate was 87 deaths per 1000 live births (national: 65 deaths per 1000 live births).^18^ We analysed data from the urban municipality of Réo and the rural municipality of Godyr, in which birth registration was previously only available in principal registration centres. The Sant’Egidio birth registration programme conceived and realized the activation of secondary registration centres at the seven health centres of Réo and the four health centres of Godyr. Civil registrars, one per health centre, operated these secondary registration centres 5 days per week from February 2015 in Godyr and July 2015 in Réo. We investigated the impact on the proportion of timely registrations until April 2017 in both municipalities. 

We also obtained data on the numbers of births registered within 60 days from two comparable control municipalities (Yako and Gomponsom) located in the Nord region, where registration is only available in principal registration centres. This allowed us to assess any potential external variables that may influence birth registration trends, for example, the time of year or the occurrence of political and administrative changes.

### Registrar training

With the assistance of the ministries of Justice and Territorial Administration, the Sant’Egidio birth registration programme provided a 3-day training course for all registrars. The course included training on civil registration and procedures, the importance of raising awareness of civil registration, and on interviewing consenting mothers and recording their personal data. At health centres, registrars approached mothers and informed of the availability of the registration office and invited the mothers to be interviewed. However, some mothers left the health centres without meeting registrars, due to closed register or short visit. During the interviews, registrars recorded on paper whether a mother was a French speaker.

### Data sources

The numbers of timely birth registrations at principal and secondary registration centres were collected during January 2014–April 2017 for the study municipalities of Réo and Godyr, and for the period January 2014–June 2016 for the control municipalities of Yako and Gomponsom. Delivery and vaccination data from the health centres hosting secondary registration centres were acquired for the study municipalities for the approximate 2-year period until April 2017. 

We digitalized and integrated paper-based data from the civil and health registers and from the interviews. We eliminated obvious recording errors, such as improbable birth dates, by manually linking records at the individual level with deliveries and vaccinations retrieved from health registers; we did not use any software to correct for missing or false data. In the event of data discrepancies, we considered civil registers to be the most accurate source. 

**Fig. 1:**
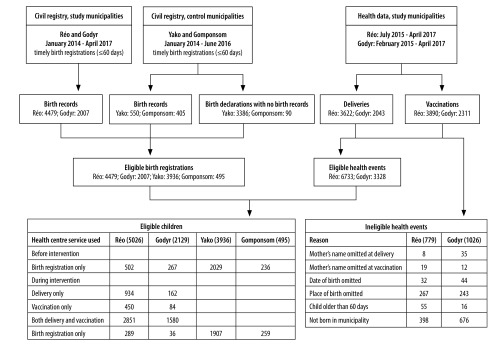
Numbers of birth registrations, deliveries, vaccinations and eligible children in the study and control municipalities

Note: birth records: births recorded in the official register of the municipality; birth declarations: births attested by a paper note in the registration office and a birth certificate released to the person, but not recorded in the official register due to administrative shortcomings; and birth registrations: total of birth records + birth declarations.

We considered all children recorded in the civil or health registers to be eligible for the impact evaluation, except for 779 records in Réo and 1026 in Godyr (Fig. 1). 

Data from the control municipalities sometimes included incomplete registrations, referred to as “birth declarations with no birth record.” In these cases, birth certificates were given to the parents and the corresponding pages of the registers were left blank; an attached note reported the birth declaration. Both birth records and birth declarations were included in the comparisons between study and control municipalities.

### Data processing

To determine the impact of our intervention, we compared the numbers of timely birth registrations before and after the introduction of the secondary registration centres. To compare municipalities with different population sizes we calculated the number of timely birth registrations per 1000 population (no. registered births × 1000/population). To consider seasonality, we analysed data from the 12 months leading to the start of the intervention and the first 12 months of the intervention.

We estimated the populations of Réo and Godyr in 2015, the middle year of the study, by applying the annual growth rates for Burkina Faso to the 2006 census data,^29^ which reported that Réo had 62 208 inhabitants and Godyr 19 320. From average annual population growth rates provided by the United Nations Population Division^30^ for Burkina Faso of 3.01% and 2.98% for the periods 2005–2010 and 2010–2015, respectively, the populations of Réo and Godyr in 2015 were estimated at 81 120 and 25 194.

To calculate the proportion of birth registration we first divided the number of timely birth registrations by the number of newborns. To estimate the crude number of newborns, we calculated the expected number of births applying the birth rate of 4.12 births per 1000 inhabitants at regional level, according to 2010 Demographic and Health Surveys,^18^ to the estimated population for 2015, resulting in 3342 expected births in Réo and 1038 in Godyr. However, this crude estimation introduced too much uncertainty. We therefore estimated a more accurate number of children born in the two municipalities, by obtaining data on all newborns attending health centres, since only 72.9% (6794/9324) of newborns in the health district of Réo and Godyr were delivered at a health centre.^31^ Almost all newborns were brought to a health centre for pentavalent vaccination.^31^ We therefore defined proportions of civil registered children as the number of registered births divided by the number of eligible infants.

### Statistical analyses

We anonymized all data before conducting statistical analyses. We performed comparisons between timely registered births in the study and control municipalities, as appropriate, with the two-sample *t-*test and the Pearson *χ^2^*-test. We used a logistic regression model to study the association between the probability of a birth not being registered within 60 days and demographic and health characteristics of the mother and child. The binary outcome variable was birth registration (no: 1; yes: 0). As predictors, we included which health centre services had been used (delivery, vaccination, delivery and vaccination, or only birth registration), mother’s characteristics (age, ethnicity and if a French speaker), birth characteristics (sex and month of birth) and the distance between the health centre and usual residence. We used the Wald *χ^2^*-test and the likelihood-ratio test to assess the partial effect of predictors and the goodness of fit.

We only included parity, weight of mother (classified as underweight, normal weight or overweight by calculating body mass index) and any problems at delivery in the models for Godyr. Such data were either unreliable (in the case of parity) or had only been partially recorded in the registers of the Réo health centres (mother’s weight status and whether problems at delivery were not recorded for 98.4% and 97.7% of mothers, respectively). Parity information was obtained either during the interview with the mother or extracted from the register for delivery. Whether any delivery problems were experienced was considered as a binary variable: either no problems (0), or a preterm birth, resuscitated child or perinatal death (1).

*P*-values of less than 0.05 were considered statistically significant. Statistical analyses were performed using SAS software 9.3 (SAS Institute, Cary, United States of America).

### Ethics

The *Comité d’ethique pour la recherche en santé* of Burkina Faso and our institutional review boards all approved our study procedures.^32^ Mothers who consented to be interviewed signed a declaration allowing the processing of their personal data for research purposes.

## Results

Of the 7512 deliveries and vaccinations recorded in the health registers of Réo, 779 (10.4%) were not eligible for the impact evaluation; of the 4354 recorded in the health registers of Godyr, 1026 (23.6%) did not meet the eligibility criteria (Fig. 1). During the first 12 months of the intervention, 2094 out of 2433 eligible births were registered within 60 days in Réo (86.1%) and 793 out of 829 in Godyr (95.7%; Table 1).

**Table 1:** Numbers of timely birth registrations according to demographic and health characteristics of mothers and infants in Burkina Faso, for a 12-month period from February 2015 (Godyr) and July 2015 (Réo)

Covariate	Réo				Godyr		
	No. of births (%)^a^		Registered births of eligible infants (%)^b^		No. of births (%)^a^		Registered births of eligible infants (%)^b^
	Eligible (*n* = 2433)	Registered (*n* = 2094)			Eligible (*n* = 829)	Registered (*n* = 793)	
**Health centre service used**							
Delivery only	426 (17.5)	294 (14.0)	69.0		57 (6.9)	40 (5.0)	70.2
Vaccination only	227 (9.3)	212 (10.1)	93.4		41 (4.9)	34 (4.3)	82.9
Both delivery and vaccination	1665 (68.4)	1473 (70.3)	88.5		725 (87.5)	713 (89.9)	98.3
Birth registration only	115 (4.7)	115 (5.5)	100.0		6 (0.7)	6 (0.8)	100.0
**Month of birth**							
January	205 (8.4)	157 (7.5)	76.6		89 (10.7)	83 (10.5)	93.3
February	146 (6.0)	121 (5.8)	82.9		88 (10.6)	80 (10.1)	90.9
March	154 (6.3)	141 (6.7)	91.6		62 (7.5)	60 (7.6)	96.8
April	196 (8.1)	189 (9.0)	96.4		64 (7.7)	63 (7.9)	98.4
May	219 (9.0)	206 (9.8)	94.1		60 (7.2)	58 (7.3)	96.7
June	223 (9.2)	197 (9.4)	88.3		63 (7.6)	59 (7.4)	93.7
July	151(6.2)	128 (6.1)	84.8		54 (6.5)	52 (6.6)	96.3
August	218 (9.0)	179 (8.5)	82.1		74 (8.9)	72 (9.1)	97.3
September	252 (10.4)	207 (9.9)	82.1		69 (8.3)	66 (8.3)	95.7
October	251 (10.3)	195 (9.3)	77.7		65 (7.8)	63 (7.9)	96.9
November	218 (9.0)	196 (9.4)	89.9		81 (9.8)	79 (10.0)	97.5
December	200 (8.2)	178 (8.5)	89.0		60 (7.2)	58 (7.3)	96.7
**Sex of baby**							
Male	1217 (50.0)	1053 (50.3)	86.5		408 (49.2)	392 (49.4)	96.1
Female	1207 (49.6)	1041 (49.7)	86.2		421 (50.8)	401 (50.6)	95.2
Not recorded	9 (0.4)	0 (0.0)	0.0		0 (0.0)	0 (0.0)	0.0
**Mother’s age, years**							
< 18	46 (1.9)	45 (2.1)	97.8		40 (4.8)	39 (4.9)	97.5
18–19	137 (5.6)	136 (6.5)	99.3		46 (5.5)	45 (5.7)	97.8
20–24	419 (17.2)	413 (19.7)	98.6		134 (16.2)	132 (16.6)	98.5
25–29	397 (16.3)	391 (18.7)	98.5		153 (18.5)	151 (19.0)	98.7
30–34	332 (13.6)	331 (15.8)	99.7		124 (15.0)	122 (15.4)	98.4
35–39	206 (8.5)	203 (9.7)	98.5		68 (8.2)	67 (8.4)	98.5
≥ 40	104 (4.3)	102 (4.9)	98.1		45 (5.4)	44 (5.6)	97.8
Not recorded	792 (32.6)	473 (22.6)	59.7		219 (26.4)	193 (24.3)	88.1
**Parity^c^**							
1	1650 (67.8)	1330 (63.5)	80.6		221 (26.7)	208 (26.2)	94.1
2	275 (11.3)	266 (12.7)	96.7		122 (14.7)	117 (14.8)	95.9
3	165 (6.8)	162 (7.7)	98.2		109 (13.1)	108 (13.6)	99.1
4	157 (6.5)	154 (7.4)	98.1		121 (14.6)	119 (15.0)	98.3
5	83 (3.4)	82 (3.9)	98.8		96 (11.6)	93 (11.7)	96.9
6	54 (2.2)	54 (2.6)	100.0		67 (8.1)	67 (8.4)	100.0
7	37 (1.5)	34 (1.6)	91.9		46 (5.5)	39 (4.9)	84.8
≥ 8	12 (0.5)	12 (0.6)	100.0		47 (5.7)	42 (5.3)	89.4
**Mother’s ethnicity**							
Gourounsi	2145 (88.2)	1915 (91.5)	89.3		624 (75.3)	604 (76.2)	96.8
Mossi	116 (4.8)	97 (4.6)	83.6		157 (18.9)	155 (19.5)	98.7
Other	20 (0.8)	20 (1.0)	100.0		26 (3.1)	26 (3.3)	100.0
Not recorded	152 (6.2)	62 (3.0)	40.8		22 (2.7)	8 (1.0)	36.4
**Mother a French speaker**							
Yes	434 (17.8)	422 (20.2)	97.2		133 (16.0)	131 (16.5)	98.5
No	1220 (50.2)	1179 (56.3)	96.6		610 (73.6)	596 (75.2)	97.7
Not recorded	779 (32.0)	493 (23.5)	63.3		86 (10.4)	66 (8.3)	76.7
**Mother interviewed**							
Yes	1845 (75.8)	1763 (84.2)	95.6		800 (96.5)	782 (98.6)	97.7
No	416 (17.1)	224 (10.7)	53.8		26 (3.1)	10 (1.3)	38.5
Not recorded	172 (7.1)	107 (5.1)	62.2		3 (0.4)	1 (0.1)	33.3
**Usual residence**							
Outside the municipality	248 (10.2)	157 (7.5)	63.3		227 (27.4)	217 (27.4)	95.6
> 10 km	29 (1.2)	24 (1.1)	82.8		27 (3.3)	25 (3.1)	92.6
5–10 km	0 (0.0)	0 (0.0)	–		233 (28.1)	219 (27.6)	94.0
< 5 km	2156 (88.6)	1913 (91.4)	88.7		342 (41.3)	332 (41.9)	97.1
**Mother’s weight status^d^**							
Underweight	3 (0.1)	3 (0.1)	100.0		15 (1.8)	15 (1.9)	100.0
Normal weight	19 (0.8)	19 (0.9)	100.0		453 (54.6)	439 (55.4)	96.9
Overweight	16 (0.7)	16 (0.8)	100.0		97 (11.7)	91 (11.5)	93.8
Not recorded	2395 (98.4)	2056 (98.2)	85.8		264 (31.8)	248 (31.3)	93.9
**Problems at delivery**							
Yes	9 (0.4)	1 (0.1)	11.1		24 (2.9)	14 (1.8)	58.3
No	46 (1.9)	45 (2.1)	97.8		646 (77.9)	631 (79.6)	97.7
Not recorded	2378 (97.7)	2048 (97.8)	86.1		159 (19.2)	148 (18.7)	93.1

^a^ Percentages do not always add up to 100.0% due to rounding errors.

^b^ Number of timely registered births as a percentage of the number of eligible births.

^c ^Registration of most recently-borne child only.

^d ^Mother’s weight was classified by calculating body mass index from measured weight and height.

### Changes in registration

**Fig. 2:**
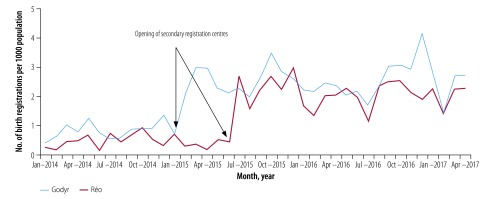
Number of birth registrations per 1000 population in the study municipalities of Réo and Godyr, Burkina Faso, January 2014–April 2017

Comparisons between the first 12 months of the intervention and the previous 12 months in Réo and Godyr indicate an increase in the number of timely registrations from 502 to 2094 in Réo (317.1%) and from 267 to 793 in Godyr (197.0%). After the introduction of secondary registration centres, birth registrations in Réo (from July 2015) and Godyr (from February 2015) followed similar seasonal trends (Fig. 2); both were characterized by low points that coincided with severe weather conditions, the Harmattan wind (February) and intense rainfalls (June–July), followed by registration peaks. However, no significant fluctuations were observed in connection with major political upheavals, namely the insurrection and deposition of the president and the subsequent removal of all mayors (October to December 2014), a coup attempt and political elections (September to November 2015) and administrative elections (May 2016).

**Fig. 3:**
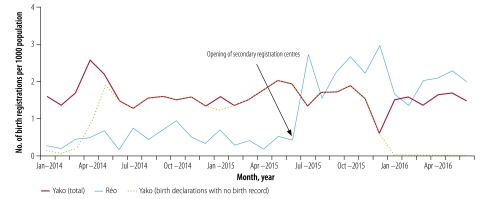
Number of birth registrations per 1000 population in the urban municipalities of Réo (study) and Yako (control), Burkina Faso, January 2014–June 2016

Note: In 2015, the estimated population was 81 120 in Réo and 105 529 in Yako.

**Fig. 4:**
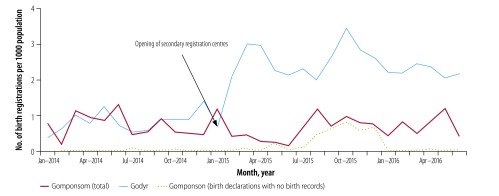
Number of birth registrations per 1000 population in the rural municipalities of Godyr (study) and Gomponsom (control), Burkina Faso, January 2014–June 2016

Note: In 2015, the estimated population was 25 194 in Godyr and 23 822 in Gomponsom.

Comparing the trends in timely birth registration showed that before the intervention, the numbers of birth registrations per 1000 population were lower in Réo than in the control municipality Yako; however, these increased in Réo after opening secondary registration centres (Fig. 3). The level of birth registrations in Godyr and Gomponsom were similar before the intervention and increased in Godyr after the implementation of the intervention (Fig. 4). Further, all birth declarations in Réo and Godyr matched the official birth records, while 86.0% (3386/3936) of declarations in Yako and 18.2% (90/495) in Gomponsom were not converted into valid birth records (Fig. 1).

Before the intervention, the mean period between birth and registration was 19.5 ± 15.3 days in Réo and 25.3 ± 18.5 days in Godyr. During the intervention, this period was significantly reduced to 10.8 ± 14.4 days (*P* < 0.0001) and 7.3 ± 11.3 days (*P* < 0.0001), respectively.

Over the same interval, the mean period between birth and registration did not change significantly in control municipalities: from 27.3 ± 18.6 days to 24.8 ± 16.0 days in Yako (*P* = 0.339), and from 23.2 ± 55.7 days to 21.3 ± 18.3 days in Gomponsom (*P* = 0.745). 

Overall, 19.6% of the children in Réo (410/2094) and 31.5% in Godyr (250/793) were registered on the day of delivery or the day of vaccination.

### Factors affecting registration

During the first year of the intervention, 68.4% (1665/2433) of newborns in Réo and 87.5% (725/829) in Godyr were delivered and vaccinated in a health centre; among these infants, the percentages of timely civil registrations were 88.5% (1473/1665) and 98.3% (713/725), respectively. Considering newborns who received vaccinations only, the births of 93.4% (212/227) in Réo and 82.9% (34/41) in Godyr were formally registered within 60 days. Of newborns delivered in health centres, but who did not receive vaccinations, the births of 69.0% (294/426) in Réo and 70.2% (40/57) in Godyr were formally registered (Table 1). No significant differences in the proportion of birth registrations were observed in relation to mother’s ethnicity, age, parity, and whether a normal weight or a French speaker.

We developed two basic logistic models for Réo and Godyr to test for the probability of a birth being registered within 60 days during the intervention (Table 2). In both municipalities, the infants of mothers attending health centres only for delivery had a higher probability of not being formally registered than those attending for vaccination or for delivery and vaccination. In both municipalities, the infants of mothers who were not interviewed had a higher probability of not being formally registered. Distance between usual residence and the health centre service used influenced whether the birth was registered in Réo, but not in Godyr. The month of birth appeared to be statistically significant in Réo, but not in Godyr; however, this result was probably due to the smaller population size in Godyr. 

**Table 2:** Risk of birth not being registered within 60 days according to demographic and health characteristics of mothers and infants in Burkina Faso, for a 12-month period from February 2015 (Godyr) and July 2015 (Réo)

Covariate	OR (95% CI)			
	Réo		Godyr	
	Basic model		Basic model	Basic model incorporating health characteristics of mother and infant
**Health centre service used**				
Delivery only	2.83 (1.82–4.41)		34.48 (9.71–125.00)	18.18 (4.51–76.92)
Vaccination only, delivery and vaccination, or birth registration only	1.00		1.00	1.00
**Month of birth**				
January	5.68 (2.32–14.09)		1.12 (0.10–12.50)	1.14 (0.08–15.87)
February	2.91 (1.13–7.52)		3.86 (0.37–40.00)	4.18 (0.34–50.00)
March	1.43 (0.50–4.07)		2.87 (0.16–52.63)	3.53 (0.17–71.43)
April	0.68 (0.21–2.17)		0.44 (0.02–12.50)	0.66 (0.02–20.41)
May	1.58 (0.56–4.44)		2.54 (0.18–37.04)	4.02 (0.23–71.43)
June	4.22 (1.69–10.53)		2.74 (0.18–41.67)	2.26 (0.10–52.63)
July	2.65 (0.99–7.04)		1.62 (0.10–27.03)	1.54 (0.07–32.26)
August	2.28 (0.92–5.65)		0.29 (0.01–6.10)	0.35 (0.02–8.07)
September	2.83 (1.17–6.85)		1.70 (0.09–32.26)	3.20 (0.14–76.92)
October	4.00 (1.66–9.62)		4.74 (0.32–71.43)	5.16 (0.29–90.91)
November	0.72 (0.29–1.77)		2.09 (0.14–31.25)	1.74 (0.10–29.41)
December	1.00		1.00	1.00
**Sex of baby**				
Female	0.99 (0.67–1.45)		1.07 (0.41–2.82)	1.09 (0.39–3.04)
Male	1.00		1.00	1.00
**Mother’s age, years**				
< 18	2.86 (0.31–26.32)		1.75 (0.13–23.26)	0.79 (0.04–14.93)
18–19	0.38 (0.04–3.44)		2.38 (0.20–28.57)	1.78 (0.14–22.22)
20–24	1.00		1.00	1.00
25–29	1.31 (0.40–4.35)		0.20 (0.02–1.80)	0.33 (0.03–3.46)
30–34	0.27 (0.03–2.35)		0.74 (0.10–5.68)	1.16 (0.12–11.24)
35–39	0.74 (0.17–3.28)		1.45 (0.24–8.85)	0.87 (0.09–9.01)
≥ 40	1.72 (0.29–10.31)			
Not recorded	34.48 (14.29–83.33)		4.33 (0.90–20.83)	3.32 (0.62–17.54)
**Parity^a^**				
1–2	NA		NA	1.00
3–4	NA		NA	0.56 (0.11–2.79)
5–6	NA		NA	0.33 (0.03–3.62)
≥ 7	NA		NA	2.71 (0.45–16.39)
**Mother’s ethnicity**				
Gourounsi	1.00		1.00	1.00
Mossi and other	1.55 (0.71–3.39)		0.16 (0.02–1.11)	0.26 (0.04–1.90)
Not recorded	2.25 (1.25–4.03)		7.87 (0.81–76.92)	10.31 (0.96–111.11)
**Mother a French speaker**				
Yes	1.00		1.00	1.00
No	0.45 (0.21–1.00)		1.29 (0.23–7.19)	1.53 (0.23–10.00)
Not recorded	3.92 (1.85–8.33)		9.62 (1.41–66.67)	12.66 (1.46–111.11)
**Mother interviewed**				
Yes	1.00		1.00	1.00
No	6.25 (4.10–9.52)		25.64 (4.31–166.67)	22.22 (3.36–142.86)
**Usual residence**				
Outside the municipality	2.63 (1.58–4.39)		0.16 (0.02–1.38)	0.15 (0.02–1.42)
> 10 km	0.88 (0.16–4.81)		10.31 (0.89–125.00)	5.71 (0.37–90.91)
5–10 km	NA		2.79 (0.96–8.13)	2.05 (0.65–6.45)
< 5 km	1.00		1.00	1.00
**Mother’s weight status^b^**				
Underweight and normal weight	NA		NA	1.00
Overweight	NA		NA	0.61 (0.11–3.34)
Not recorded	NA		NA	0.65 (0.11–3.94)
**Problems at delivery**				
Yes	NA		NA	5.81 (0.80–41.67)
No	NA		NA	1.00
Not recorded	NA		NA	1.00 (0.14–6.94)

CI: confidence interval; NA: not applicable; OR: odds ratio.

^a ^Registration of most recently-borne child only.

^b^ Mother’s weight was classified by calculating body mass index from measured weight and height.

For Godyr, we developed an expanded logistic model by incorporating the health conditions of the mother and child, which might have affected birth registration. None of these covariates had a statistically significant association with timely registration (Table 2). To validate the inability of these variables to describe differences in the probability of timely registration, we used a likelihood-ratio test to compare the basic model with the expanded model. The difference between the −2 log-likelihood functions was 8.887 (148.629–139.742), and the *χ^2^* statistic was not statistically significant (*P* = 0.261).

## Discussion

Compared with the number of birth registrations during the year before the start of our intervention, we observed an increase in timely registrations of more than threefold in Réo and an almost twofold increase in Godyr; no major changes were observed in the control municipalities. This increase could be explained by the provision of registration offices in health centres habitually used by families, staffed by dedicated personnel. Several reported factors for registration (e.g. sex of baby and mother’s age, ethnicity and marital status)^19^^,^^21^^–^^23^^,^^33^^,^^34^ were not associated with the increase of timely registered births in the study municipalities.

Of all the services provided by health centres, birth registration proportions were lowest among mothers who attended for delivery, but did not return for vaccination. This lower performance might be due to poor access to health centres after birth, early infant mortality or high mobility. Poor access does not seem likely, however, given the high vaccination rates in the area.^31^ Early infant mortality is a possibility, and records of (non-registered) perinatal deaths were noted in health registers for delivery (31 deaths in Réo and 16 in Godyr) and reported by mothers during interviews (53 in Réo and 28 in Godyr). Future regulations could allow health and administrative staff, as well as traditional and religious authorities, to offer a clerical civil declaration of an unregistered event. Mobility is also a possible cause, being high in the Centre-Ouest region of Burkina Faso.^29^ In fact, the reason that so many children vaccinated in the study municipalities were not eligible for the impact evaluation was that they were born elsewhere. The mobility barrier could be addressed through amendments that allow for a declaration of birth in municipalities other than the birthplace. 

We noted that mothers who attended a health centre and were not interviewed by registrars demonstrated the lowest probability of registering their children. We also observed monthly fluctuations in registration trends, which could not be fully explained by adverse climate conditions. Further possible reasons must be investigated (e.g. whether a result of temporary organizational problems within particular health centres or whether a consequence of local events).

Our study has several limitations. First, we recorded all deliveries and/or vaccinated children at health centres; this covered the large majority of newborns, but not the whole infant population of the area. Our municipal birth rates implied from the number of eligible infants (3.0%; 2433/81 120; for Réo and 3.3%; 829/25 194 for Godyr) are lower than the national birth rates provided by the United Nations Population Division (4.1%),^30^ highlighting how some newborns who were not immunized or born to families who migrated may have been excluded from the number of eligible newborns. Second, the large volume of data not reported for some health variables (e.g. whether a mother had a normal weight or experienced problems at delivery) might have led to a non-random selection and, consequently, the misinterpretation of the role of some predictors. Third, we could not divide the population into wealth quintiles either from the registers or from interviews. Lastly, although several neonatal deaths were observed, none of these were registered by the parents either at birth or death; this confirms previous studies which have reported that neonatal deaths often go unregistered.^13^^,^^22^^–^^24^^,^^35^

We argue that a widespread registration system based in health centres already used by mothers for delivery and vaccination, with dedicated registry personnel, could considerably improve the proportion of births which are formally registered. The strengthening of civil status services during our intervention reinforced the administrative structure of the study municipalities, guaranteeing registration continuity during the political upheavals between 2014 and 2016. In contrast, control municipalities experienced operating problems due to political and administrative hardships, as shown by the high numbers of birth certificates with no birth records. Unlike studies that suggest the use of health personnel to register births,^20^^,^^36^ we believe that a civil registrar dedicated exclusively to sensitization, recording and digitization is essential to increase the registration of births, considering the registry expertise needed, the time required for computerization and the lack of health-care personnel. Although interval from birth to registration was reduced in both study municipalities, registrations mainly occurred on a different day from birth or vaccination; it may therefore be adequate for the registrar to attend the centre for a limited number of days per week. 

We believe that our system of introducing secondary registration centres also lends itself to meeting the challenge of recording deaths, laying the foundations for reliable civil registration systems. 
